# The Impact of SARS-CoV-2 (COVID-19) Pandemic on International Dermatology Conferences in 2020

**DOI:** 10.3389/fmed.2021.726037

**Published:** 2021-08-05

**Authors:** Eun Seo Ha, Ji Yeon Hong, Sophie Soyeon Lim, H. Peter Soyer, Je-Ho Mun

**Affiliations:** ^1^Department of Premedicine, Seoul National University College of Medicine, Seoul, South Korea; ^2^Department of Dermatology, Seoul National University Hospital, Seoul, South Korea; ^3^Alfred Health, Melbourne, VIC, Australia; ^4^The University of Queensland Diamantina Institute, The University of Queensland, Dermatology Research Center, Brisbane, QLD, Australia; ^5^Dermatology Department, Princess Alexandra Hospital, Brisbane, QLD, Australia; ^6^Department of Dermatology, Seoul National University College of Medicine, Seoul, South Korea

**Keywords:** COVID-19, conference, virtual, dermatology, coronavirus, pandemics, congress, SARS-CoV-2

## Abstract

To limit the spread of the SARS-CoV-2 (COVID-19) outbreak, humans have been significantly restricted in their ability to travel and interact with others worldwide. Consequently, dermatology conferences were forced to adapt to such changes. The aim of this study is to investigate the impact of COVID-19 on international dermatology conferences. We retrospectively investigated decisions made for international dermatology conferences scheduled for 2020. Thirty-three major conferences were analyzed. Their data were obtained from their respective websites (data was accessed 2 June 2021). Among 33 conferences analyzed, 13 (39.4%) were conducted as scheduled, nine (27.3%) were canceled, eight (24.3%) were postponed to 2021 or 2022, and three (9.1%) were delayed but conducted in 2020. The number of the cancellation (44.4%) and postponement (75%) was the largest in the second quarter of the year. During the fourth quarter, most conferences were held on schedule (70%) but were run virtually. Eight out of 13 virtual conferences shortened their duration (61.5%). Most (90.9%) conferences have decided on the schedule of their meetings for 2021 or 2022 while three (9.1%) remain undecided. Twelve (40%) are planned to run virtually, eight (26.7%) have opted for a hybrid form, five (16.7%) are planned to run in-person, four (13.3%) have not decided on the format, and one (3.3%) has been canceled. Virtual and hybrid conference formats have facilitated people to share knowledge despite the travel restrictions posed by the COVID-19 pandemic. Such formats are environmentally friendly, are able to attract a large audience, and save delegates time and costs involved in attending. Therefore, virtual platforms should continue to be integrated within conferences in the post-pandemic era.

## Introduction

The novel coronavirus disease 2019 (COVID-19) forced innumerable events to make significant adjustments in 2020 (1). Cancellation, postponement or online reformatting of major events, such as the Olympics and major film festivals, prevented expected attendees from participating in-person.

International conferences facilitate thousands of delegates to meet and discuss ideas and update each other on their topics of interest (2). However, as gatherings and international travel significantly increase the risk of viral transmission, governments worldwide have implemented strict quarantine and social distancing restrictions. Consequently, conferences have been forced to cancel, postpone or reformat their meetings in order to protect their attendees and local communities (3, 4). International dermatology conferences were no exception. In this study, we analyzed the decisions made due to COVID-19 by the major dermatological conferences regarding their meetings scheduled for 2020.

## Methods

### Study Design

We searched for international dermatology conferences arranged in 2020. Thirty-three major conferences were analyzed. Their data were obtained from their respective websites. Data collected included the venue, originally planned date, decision on cancellation or postponement and relevant details, original meeting format (in-person or virtual), change in meeting duration, date and format of the next meeting [in-person, virtual or hybrid (mix of in-person and virtual)], and the number of confirmed and deceased COVID-19 cases during 2020 in the host country (Table 1, last accessed 2 June 2021).

**Table 1:** Summary of major dermatology conferences in 2020.

	**Conference**	**City and country**	**Original date**	**Decision**	**Details**	**Meeting format**	**Period shortened**	**Next meeting schedule**	**Next meeting format**	Confirmed cases^**^ (Deceased)
1	Melanoma 2020: 30th Annual Cutaneous Malignancy Update	San Diego, USA	24–26 January	Conducted on original date		In-person	No	23–24 January 2021	Virtual	19,346,790 (335,789)
2	Maui Derm for Dermatologists 2020	Maui, Hawaii	25–29 January	Conducted on original date		In-person	No	25–29 January 2021	Hybrid	21,459 (286)
3	International Master Course on Aging Science (IMCAS) World Congress 2020	Paris, France	30 January−1 February	Conducted on original date		In-person	No	27–29 January 2022	Hybrid	2,556,708 (64,004)
4	14th International Congress of Aesthetic Dermatology (ICAD)	Bangkok, Thailand	3–4 February	Conducted on delayed date	Postponed to 20–22 November 2020 and later merged with '18th Aesthetic & Anti-aging Medicine World Congress (AMWC) Global – The Virtual Edition' and held on November 5–6, 2020	Virtual	No	18–20 November 2021	In-person^#^	6,690 (61)
5	20th Edition of Dubai World Dermatology and Laser conference (Dubai Derma)	UAE, Dubai	16–18 March	Canceled^*^	Postponed initially to 16–18 June 2020 and later to 2–4 March 2021 and finally to 6–8 July 2021	-	No	6–8 July 2021	Hybrid	206,092 (665)
6	2020 Annual Meeting of the American Academy of Dermatology (AAD)	Denver, USA	19–23 March	Canceled		-		Canceled	Canceled	19,346,790 (335,789)
7	16th European Association of Dermato-Oncology (EADO) Congress	Vilnius, Lithuania	20–25 April	Conducted on delayed date	held on 12–14 October 2020	Virtual	Yes (3 days)	15–17 April 2021	Virtual	140,579 (1,458)
8	International Society of Atopic Dermatitis (ISAD) 2020	Seoul, South Korea	22–24 April	Postponed to 2021	postponed to 19–20 April 2021 and provided 3 hours of live streaming on 3 September 2020 instead	-	Yes (1 day)	19–20 April 2021	Hybrid	60,734 (900)
9	7th Continental Congress of Dermatology (CCD)	Mexico City, Mexico	22–25 April	Canceled		-		2022 (Final dates to be determined)	ND^#^	1,401,529 (123,845)
10	16th spring symposium of the European Academy of Dermatology and Venereology (EADV)	Poro, Portugal	30 April−2 May	Canceled		-		20–22 May 2021	Virtual	406,051 (6,830)
11	38th Annual Meeting of Latin American Dermatologists (RADLA)	Asunción, Paraguay	1–4 May	Postponed to 2021	Postponed to 15–18 April 2021	-	No	15–18 April 2021	Virtual	106,136 (2,220)
12	2nd World Congress of Trichoscopy	Sorrento, Italy	6–8 May	Postponed to 2021	Postponed to 9–11 October 2021	-	No	9–11 October 2021	ND	2,083,689 (73,604)
13	53rd Annual Meeting of the Australian College of Dermatologists (ACDASM)	Adelaide, Australia	6–9 May	Postponed to 2021	Postponed to 9–11 April 2021	-	Yes (1 day)	9–11 April 2021	Virtual	28,381 (909)
14	20th European Society for Pediatric Dermatology (ESPD) Annual Meeting	Vienna, Austria	11–13 May	Postponed to 2021	Postponed to 12–14 May 2021	-	No	12–14 May 2021	Virtual	356,351 (6,086)
15	78th Annual Meeting of the Society for Investigative Dermatology (SID)	Arizona, USA	13–16 May	Conducted on original date	Free virtual meeting of selected content of the original program	Virtual	No	3–8 May 2021	Virtual	19,346,790 (335,789)
16	15th World Congress of International Academy of Cosmetic Dermatology (IACD)	Dresden, Germany	18–20 June	Canceled	Postponed initially to 1–3 July 2021 and later canceled	-	No	ND	ND	1,719,737 (33,071)
17	24th International Pigment Cell Conference (IPCC)	Yamagata, Japan	18–21 June	Canceled		-		May/June 2023(Final dates to be determined)	ND	230,304 (3,414)
18	18th World Congress on Cancers of the Skin (WCCS)	Buenos Aires, Argentina	24–27 June	Postponed to 2021	Postponed initially to 3–6 November 2021 and later to 2–5 November 2022	-	No	2–5 November 2022	ND	1,602,163 (43,018)
19	100th Annual Meeting of the British Association of Dermatologists (BAD)	Manchester, UK	7–9 July	Conducted on delayed date	Held on 1–3 September 2020	Virtual	No	6–8 July 2021	Virtual	2,432,892 (72,548)
20	45th Annual Meeting Society for Pediatric Dermatology Annual meeting (SPDA)	Asheville, USA	9–12 July	Conducted on original date	Held on 10–12 July 2020	Virtual	Yes (1 day)	8–10 July 2021	Virtual	19,346,790 (335,789)
21	72nd Annual Meeting of the Pacific Dermatologic Association (PacDerm)	San Francisco, USA	30 July−2 August	Canceled		-		19–22 August 2021	Hybrid	19,346,790 (335,789)
22	American Academy of Dermatology (AAD) 2020 Summer Meeting	Seattle, USA	13–16 August	Canceled		-		5–8 August 2021	In-person	19,346,790 (335,789)
23	12th 5-Continent-Congress (5CC) World Congress	Barcelona, Spain	27–30 August	Conducted on original date	Held on 28–30 August 2020	Virtual	Yes (1 day)	4–5 September 2021	Virtual	1,893,502 (50,442)
24	American Society for Dermatologic Surgery (ASDS) Annual Meeting 2020	Washington DC, USA	8–11 October	Conducted on original date	Held on 9–11 October 2020	Virtual	Yes (1 day)	14–17 October 2021 (In person) 19–21 November 2021(Virtual)	Hybrid	19,346,790 (335,789)
25	41st Annual Meeting of the International Society for Dermatologic Surgery (ISDS)	Cairo, Egypt	20–24 October	Conducted on original date	Held on 21–23 October 2020	Virtual	Yes (1 day)	ND	ND	136,644 (7,576)
26	Australasian Melanoma Conference (AMC) 2020	Sydney, Australia	23–24 October	Canceled^*^	Postponed to 19–20 November 2021	-	No	19–20 November 2021	In-person	28,381 (909)
27	29th Congress of the European Academy of Dermatology and Venereology (EADV)	Vienna, Austria	28 October−1 November	Conducted on original date	Held on 29–31 October 2020	Virtual	Yes (2 days)	29 September-2 October 202113-17 October 2021	Virtual	356,351 (6,086)
28	17th International Congress of the Society for Melanoma Research (SMR)	New Orleans, USA	29 October−1 November	Conducted on original date	Held on 28 October 2020	Virtual	Yes (3 days)	28–31 October 2021	Hybrid	19,346,790 (335,789)
29	8th World Congress of Teledermatology, Imaging, and Artificial Intelligence for Skin Diseases	Seville, Spain	5–6 November, 2020	Conducted on original date	Held on 5–6 November, 2020	Virtual	No	ND	ND	1,893,502 (50,442)
30	57th American Society of Dermatopathology (ASDP) Annual Meeting	Chicago, USA	5–8 November	Conducted on original date	Held on 5–11 November 2020	Virtual	Lengthened (3 days)	20–24 October 2021	Virtual	19,346,790 (335,789)
31	9th Dermatologic & Aesthetic Surgery International League (DASIl) World Congress	Mexico City, Mexico	11–14 November	Conducted on original date	Held on 13–15 November 2020	Virtual	Yes (1 day)	27–30 October 2021	In-person	1,401,529 (123,845)
32	4th Inflammatory Skin Disease Summit (ISDS)	New York, USA	18–21 November	Postponed to 2021	Postponed to 3–6 November 2021	-	No	3–6 November 2021	Hybrid	19,346,790 (335,789)
33	6th Eastern Asia Dermatology Congress (EADC)	Gyeongju, South Korea	25–27 November	Postponed to 2022	Initially delayed to 7–9 July 2021 and later delayed to 8–10 December 2022	-	No	8–10 December 2022	In-person	60,734 (900)

* *When the annual conference which always takes the same venue was postponed one year, we classified it as “Cancelled” even though it announced postponement. When the conference, which rotates its venue internationally, is postponed for more than one year, we marked it as “Postponed to next years (2021 or 2022)” as long as the venue is still the same*.

** *Confirmed and deceased cases refer to those of the host countries; #ND stands for “not determined”*.

For the conferences of which the upcoming meeting dates are confirmed, their final schedules are described in Table 1. We applied “Not Determined (ND)” for the undecided schedules. The format of subsequent meetings was also marked as “ND” if the conferences did not decide whether they would be in-person, virtual or hybrid. Furthermore, we recorded the official World Health Organization (WHO) reported total number of confirmed and deceased COVID-19 cases in the hosting countries until December 31, 2020. This study was exempted from ethics review as it investigated publicly available data.

### Classification of the Decision Made

We classified the decisions made for each conference into four categories: “Conducted on original date”, “Conducted on delayed date”, “Postponed (to 2021 or 2022)”, or “Cancelled”. “Conducted on original date” refers to the meetings that were hosted on the originally planned date. “Conducted on delayed date” stands for the conferences that were held on delayed dates within 2020. If an annual conference which is always held in one country is postponed a year, it was classified as “Cancelled”. Indefinitely postponed conferences were classified as “Cancelled” as well. The 20th Edition of Dubai World Dermatology and Laser conference and the Australasian Melanoma Conference 2020 (6th and 27th on Table 1) were such cases. However, if a conference which is held in a different country each time is postponed for >1 year, it was marked as “Postponed (to 2021 or 2022)” as long as the hosting country remained the same.

## Results

### Overall Fate of Conferences in 2020

Out of the 33 international dermatology conferences, 13 (39.4%) were conducted as scheduled, nine (27.3%) were canceled, eight (24.3%) were postponed to 2021 or 2022, and three (9.1%) were delayed but conducted in 2020. Among 16 meetings held in 2020, 13 (81.25%) were held virtually, and three (18.75%) were held in-person. All in-person meetings were held in January 2020.

### Change in Conference Schedules per Quarter

In each quarter of 2020, 6, 12, 5, and 10 conferences were originally scheduled to be held. Out of the conferences scheduled January–March, three (50%) were conducted on their original dates, two (33.3%) were canceled and one (16.6%) was conducted on delayed dates. Out of the conferences scheduled April–June second quarter, six (50%) were postponed, four (33.3%) were canceled, one was (8.3%) conducted on its original date and one (8.3%) was delayed but conducted in 2020. Out of the conferences scheduled July–September, two (40%) were conducted on their original dates, two (40%) were canceled and one (20%) was conducted on a delayed date. Out of the conferences scheduled October–December, seven (70%) were conducted on their original dates, two (20%) were postponed and one (10%) was canceled.

The number of conferences conducted on their original dates was the largest in the fourth quarter of the year (53.8%), followed by the first quarter (23.1%), the third quarter (15.4%) and the second quarter (7.7%). The number of canceled conferences was the largest in the second quarter (44.4%), followed by third (22.2%), first (22.2%) and fourth (11.1%). COVID-19 appears to have had the most significant impact on conferences in the second quarter of 2020, when the World Health Organization (WHO) declared it as a pandemic. All the conferences held on their original dates in the fourth quarter were held virtually. Three conferences which were delayed but conducted in 2020 ran virtually.

### Duration of Conferences Held

Of the 16 completed meetings, eight (50%) were shortened, seven (43.75%) were conducted as scheduled and one (6.25%) was lengthened. All in-person conferences in January 2020 were run as scheduled, while most virtual meetings (eight out of 13, 61.5%) shortened their duration (the original duration: 3.70 ± 0.9, the changed duration: 3.31 ± 1.1 days, mean ± standard deviation).

### Decision on Upcoming Meetings

Most (93.9%) conferences have decided on the schedule of their meetings for 2021 or 2022 while two (3.1%) remain undecided. Of the 31 arranged conferences, 12 (38.7%) are planned to run virtually, eight (25.8%) have opted for a hybrid format, five (16.1%) are planned to run in-person, four (12.9%) are undecided, and two (6.5%) have been canceled. Among nine meetings scheduled for the first half of 2021, seven (77.8%) were virtual and two (22.2%) were hybrid. Of the 15 conferences scheduled for the second half of 2021, five (33.3%) are scheduled to run virtually, five (33.3%) have opted for a hybrid format, four (26.7%) are planned to run in-person and one (6.7%) remains undecided.

## Discussion

In this study, we analyzed how major dermatology conferences in 2020 adapted to restrictions set by the COVID-19 pandemic. The results denote close association between the date and the decisions made by the conferences. The WHO declared the outbreak of COVID-19 on January 30, 2020. Before the declaration, all three conferences in January were conducted in-person. However, all 30 conferences planned from February to December 2020 were canceled, rescheduled or switched to virtual form.

COVID-19 had the most significant impact on conferences during the second quarter of 2020 when the WHO declared it as a pandemic. Eleven out of 12 conferences planned for the second quarter deferred or canceled their meetings. The second quarter accounted for the largest percentage of cancellations (44.4%) among four quarters. In contrast, seven out of 10 meetings originally scheduled for the fourth quarter were run as scheduled but in virtual form. These results show how the conference hosts with meetings scheduled near the end of the year had sufficient time to re-organize and run their events virtually to adapt to COVID-19 restrictions. Success of these virtual conferences was enabled by audio-visual e-platforms, such as Zoom (Zoom Video Communications, California, U.S.), Cisco Webex (Cisco Webex, California, U.S.) and Google Hangout (Google, California, U.S.), which developed rapidly due to the exponential increase in demand during the pandemic.

Running conferences virtually has several advantages and disadvantages (Table 2). The greatest advantage is that virtual conferences have significant flexibility in timing and location. Thus, they can host a much larger number of attendees compared to in-person conferences, thereby offering economy of scale to reduce registration fees (7). Running conferences virtually may be more profitable for the hosting organizations as they may reduce costs on venue hire and staffing (8). Affordable fees can make meetings more accessible for a larger audience (9). Talks may even be recorded and transmitted via delayed streaming to let attendees choose their best time to view the lecture. (5) In addition, virtual conferences are more environmentally friendly as they can reduce the carbon footprint of traveling (10, 11). Finally, given that much of dermatology is image-based, it is well suited to this virtual method of knowledge distribution. (12).

**Table 2:** Advantages and disadvantages of running conferences virtually.

**Advantages**	**Disadvantages**
Allow moderators to better control the flow of sessions	Cannot conduct hands-on learning
Can record talks for future reference	Have technical issues: weak internet connection, poor audio and video quality
Can host a large number of attendees	Limit mentorship and interaction between experts and residents or students
Can allow delegates to attend regardless of location	Lose human contact, affections and emotions (5)
Reduce carbon footprint of meeting travel (6)	Lose networking opportunities amongst delegates
Reduce or eliminate registration fees becoming more affordable	Restrict interaction with dermatology-related industries (e.g., cosmetics, laser, pharmaceutical)
Reduce costs for hosting organizations (e.g., venue hire, staffing)	Prevent speakers from sufficiently engage with audience
Save travel and accommodation costs for delegates	Prevent delegates of developing countries with poor internet connection from participating

However, in-person conferences have characteristics which cannot be mimicked by virtual means. In-person conferences allow attendees of all career stages to interact, share ideas and learn from one another (5, 13). Furthermore, virtual conferences cannot provide opportunities for hands-on learning, such as dermatologic surgery skills, or mentorship between leading dermatology experts and junior doctors as well as students. Virtual meetings are, by nature, not as engaging as in-person interactions, as audience members are reduced to names on screens. In our data, alteration to virtual format was accompanied by shortening of conference duration in the majority of conferences. The average conference duration decreased from 3.7 to 3.3 days. This may be due to the deletion of activities only feasible in-person, such as workshops and welcome receptions, which further reduces networking opportunities for attendees.

Technical issues including intermittent connection, poor audio and video quality significantly disrupt delegates' ability to distribute information and interact with one another (14). Hyper-efficient telecommunication networks and optimal image quality are prerequisites for real-time video and audio interaction. Therefore, attendees from underdeveloped societies may not be able to participate, leading to an imbalance of knowledge distribution and opportunities. Virtual conferences also restrict dermatology experts from interacting with dermatology-related industries, such as cosmetics, pharmaceutical and laser companies, and helping them manufacture evidence-based products.

It is likely that most conferences in 2021 will run virtually due to the COVID-19 pandemic. All nine conferences scheduled for the first half of 2021 were run virtually (77.8%) or in hybrid form (22.2%). For the second half of 2021, 10 (66.7%) out of 15 conferences are planned to be held in virtual or hybrid formats. Some organizations have even launched independent virtual conferences, rather than temporarily adopting virtual platforms to run their in-person conferences. For example, the American Academy of Dermatology (AAD) canceled their 2020 Annual Meeting. Instead, they launched the Annual Meeting of the American Academy of Dermatology Virtual Meeting Experience (AADVMX), which ran live from June 12 to 14, and academic content was made accessible until December 31, 2020. AADVMX was held from 23 to 25 April 2021 as well.

Our study has a few limitations. First, not all conferences were analyzed due to the absence of a platform that shows every dermatological conference at a glance. We however attempted to include all major gatherings that attract hundreds of attendees nationally and internationally. Second, biennial conferences which were not scheduled for 2020 but for 2021 were not included. Despite these limitations, this study sufficiently captures the effect of COVID-19 on the 2020 dermatological society and informs future decision-making for overcoming travel restrictions when organizing internationall conferences, especially when facing another pandemic.

## Conclusion

The restrictions posed by COVID-19 provided a unique opportunity for conference hosts to experiment running their events virtually. Although virtual conferences have limitations, such as technical issues and loss of networking opportunities, they allow participants across the globe to overcome physical limitations and congregate to share knowledge. Removing the need for delegates to travel long distances is also beneficial for the environment and saves delegates' time and costs when compared to attending in-person conferences. By optimizing the technicalities of virtual platforms and increasing opportunities for more liberal interaction amongst delegates, conferences should continue integrating virtual experiences for their future meetings.

## Data Availability Statement

The original contributions presented in the study are included in the article/supplementary material, further inquiries can be directed to the corresponding author/s.

## Author Contributions

EH, JH, and J-HM contributed to data acquisition and contributed to data analysis. All authors contributed equally to the concept, design, and contributed to the drafting and critical revision of the manuscript.

## Conflict of Interest

The authors declare that the research was conducted in the absence of any commercial or financial relationships that could be construed as a potential conflict of interest.

## Publisher's Note

All claims expressed in this article are solely those of the authors and do not necessarily represent those of their affiliated organizations, or those of the publisher, the editors and the reviewers. Any product that may be evaluated in this article, or claim that may be made by its manufacturer, is not guaranteed or endorsed by the publisher.
